# The Functional Impact of VX-770 on the Cystic Fibrosis Transmembrane Conductance Regulator Is Enduring and Increases the Constitutive Activity of This Channel in Primary Airway Epithelia Generated from Healthy Donors

**DOI:** 10.3390/biom14111378

**Published:** 2024-10-29

**Authors:** Heidi J. Nick, Sarah E. Christeson, Preston E. Bratcher

**Affiliations:** 1 Department of Pediatrics, National Jewish Health, Denver, CO 80206, USA; 2 Department of Pediatrics, University of Colorado Denver, Anschutz Medical Center, Aurora, CO 80045, USA

**Keywords:** cystic fibrosis, CFTR, VX-770, ivacaftor, CFTR potentiator, airway ion transport

## Abstract

VX-770 is a small-molecule CFTR potentiator that is highly efficacious in individuals with cystic fibrosis caused by mutations in CFTR that result in a defect in channel gating. While studies have reported on the mechanism of action of VX-770, there is still more to learn about the impact that it has on CFTR function in various contexts. The aim of the present study was to examine the longevity and stability of the effect of VX-770 on CFTR function in cultured airway epithelia and to measure the consequences of this interaction. The responses to acute and chronic VX-770 exposure were measured in cultures of expanded and re-differentiated primary human nasal epithelial cells. Acute VX-770 exposure resulted in an increase in CFTR-mediated currents in the absence of exogenous compounds that induce the phosphorylation/activation of CFTR, with acute exposure having the same effect as chronic exposure. The functional impact of VX-770 on CFTR was long-lasting in cultured airway epithelia, as they maintained an electrophysiological profile consistent with the saturation of CFTR with VX-770 over time periods of up to 4 days following a short (0.5 min) or low-dose (100 nM) exposure to VX-770 during an analysis in an Ussing chamber. Rinsing the apical surface prior to VX-770 exposure or exposure during the analysis in the Ussing chamber increased the interaction between VX-770 and the CFTR. Importantly, after short, low-dose exposures to VX-770, the CFTR channels in cultured epithelia appeared to remain saturated with VX-770 for extended periods of time, despite the repetitive rinsing of the apical surface. This finding has implications for patients discontinuing the use of VX-770-containing therapies.

## 1. Introduction

The introduction of VX-770 (ivacaftor, Kalydeco^©^) into clinical use for the treatment of cystic fibrosis (CF) was the first therapy targeting the underlying cause of the disease: the dysfunction of the cystic fibrosis transmembrane conductance regulator (CFTR) ion channel. As a recessive genetic disease, both the inherited alleles of CFTR must be dysfunctional in order for symptoms of CF to manifest. The symptoms resulting from impaired chloride transport across airway epithelia include the presence of thick and obstructive mucus, recurrent pulmonary infections, and decreased lung function. VX-770 is a small molecule with the ability to potentiate (increase channel gating and open probability) CFTR, and it was initially shown to be effective in improving the chloride conductance of CF-causing mutant CFTRs that have a defect in gating, including G551D and the most common mutation, F508del [1]. It was subsequently found to be effective for other CFTRs harboring gating mutations [2,3]. Clinically, the treatment of individuals with CF caused by CFTRs with gating defects or residual function has been highly efficacious, as demonstrated by improvements in lung function and decreases in sweat chloride concentrations [4,5,6,7,8].

The mechanism of the VX-770-mediated potentiation of CFTR is independent of the cAMP-mediated phosphorylation and activation of the channel, and involves bypassing the ATP-binding requirement for channel opening [9,10,11]. The functional properties of VX-770 result from its ability to directly interact with the protein and modulate channel function, and this may occur through binding to CFTR at multiple sites [10,12,13,14]. Binding is believed to result in an increased stability of the open confirmational state that occurs before ATP hydrolysis, causing an ATP-independent increase in the open probability of the channel and increasing the open time in the presence of ATP [11,15].

Several studies have reported circulating concentrations of VX-770 in people with CF undergoing VX-770 therapy, and these studies have found the maximum plasma concentration to be more than 5 μM (mean) [16,17,18]. However, as it readily binds to albumin and other serum proteins, the free active concentration of VX-770 in plasma is believed to be less than 10 nM [19]. Additionally, VX-770 concentrations have been measured in epithelial cells, where it has been shown to accumulate at concentrations more than 200-fold greater than the exposed concentration in vitro, and it can reach intracellular concentrations of over 3 μM in nasal epithelial samples obtained from individuals undergoing treatment with VX-770 [16,17,20,21]. These high intracellular concentrations of the drug raise questions about how long the drug and its effects endure after exposure has ceased.

The aim of the present study was to examine the longevity and stability of the effect of VX-770 on CFTR function in cultured airway epithelia and to measure the consequences of this interaction. This may prove valuable to understanding the length of the functional impact of drug exposure, which could be informative for short-term clinical therapies, for washing out a chronic therapeutic treatment, and for switching to a new therapy for reasons of a potential increased efficacy or enrollment in a clinical trial. The thoroughness of washout could be particularly important if there are negative interactions between VX-770 and an alternative treatment that is to be initiated.

## 2. Materials and Methods

### 2.1. Cells and Culture

Primary human nasal epithelial cells (HNECs) were obtained from brushings of the inferior turbinate using an IRB-approved protocol (HS-2832 and NGP2021-0023) and expanded using conditional reprogramming culture methods utilizing the ROCK inhibitor Y-27632 (#A3008, Apex BIO, Huston, TX, USA) and irradiated 3T3 fibroblasts, as previously described [22,23,24]. For differentiation at the air–liquid interface (ALI), the expanded cells were plated at a density of 250,000 cells/cm^2^ on 6.5 mm permeable Transwell inserts (#3270, Corning Life Sciences, Tewksbury, MA, USA) coated with bovine Type I collagen (#5005-b, Advanced BioMatrix, Carlsbad, CA, USA). They were grown submerged for 2 days in PneumaCult™-Ex Plus (#05040, STEMCELL Technologies, Vancouver, BC, Canada), and then cultured at the ALI using PneumaCult™-ALI media (#05001, STEMCELL Technologies, Vancouver, BC, Canada) for 3-5 weeks. For chronic treatment with VX-770, the cells were treated for 24 h with media containing either DMSO alone (vehicle, #BP231-100, Fisher Chemical, Pittsburgh, PA, USA) or 0.1 µM of VX-770 (#S1144, Selleck Chemicals, Huston, TX, USA) prior to the analysis in the Ussing chamber. For HNECs that were returned to the culture after the Ussing chamber analyses, the media contained 100 I.U./mL of penicillin (#30-002-CI, Corning Mediatech, Manassas, VA, USA), 100 µg/mL of streptomycin (#30-002-CI, Corning Mediatech, Manassas, VA, USA Corning), 40 µg/mL of tobramycin (NDC #0409-3578-01, Hospira, Lake Forest, IL, USA), 2 µg/mL of fluconazole (#J62015, Alfa Aesar, Haverhill, MA, USA), and 0.617 µg/mL of amphotericin B (#A9528, Sigma-Aldrich, St. Louis, MO, USA).

For apical washes, 200 µL of warm Hanks’ balanced salt solution (HBSS) with calcium and magnesium (#21-023-CV, Corning Mediatech, Manassas, VA, USA) was applied to the apical surface, and the cultures were returned to the incubator for 10 min. The apical rinse solution was then aspirated and re-applied to the culture 8 times in order to further loosen any mucus and cellular products, followed by removal by suction. These washes occurred at 90 and 60 min prior to the Ussing chamber analysis. At 30 min prior to the analysis, 100 µL of HBSS with calcium and magnesium containing either DMSO or VX-770 was applied to the apical surface, the cultures were incubated for the indicated amount of time, and the solution was removed by suction, after which the cells were rinsed twice with 200 µL of warm HBSS with calcium and magnesium.

### 2.2. Electrophysiology

**Figure 1:**
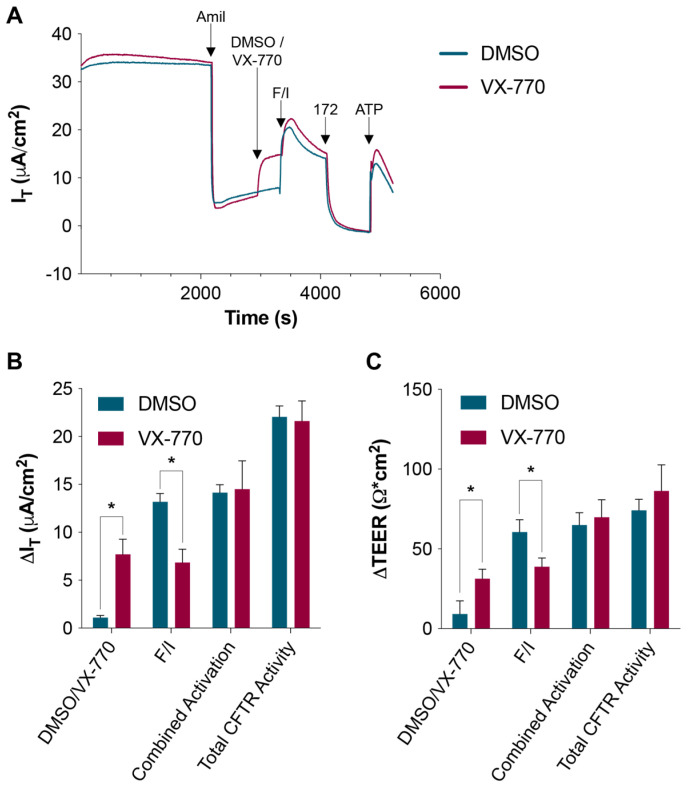
Acute exposure to VX-770 increases CFTR-mediated currents without affecting the functional capacity. (**A**) HNECs were sequentially treated with amiloride, DMSO or 1 μM VX-770, forskolin/IBMX (F/I), CFTR (inh)-172 (172), and ATP. (**B**,**C**) Changes in the transepithelial current (∆I_T_) and resistance (∆TEER) values in response to the listed compounds are presented. * *p* < 0.05. *n* = 6 per group.

**Figure 2:**
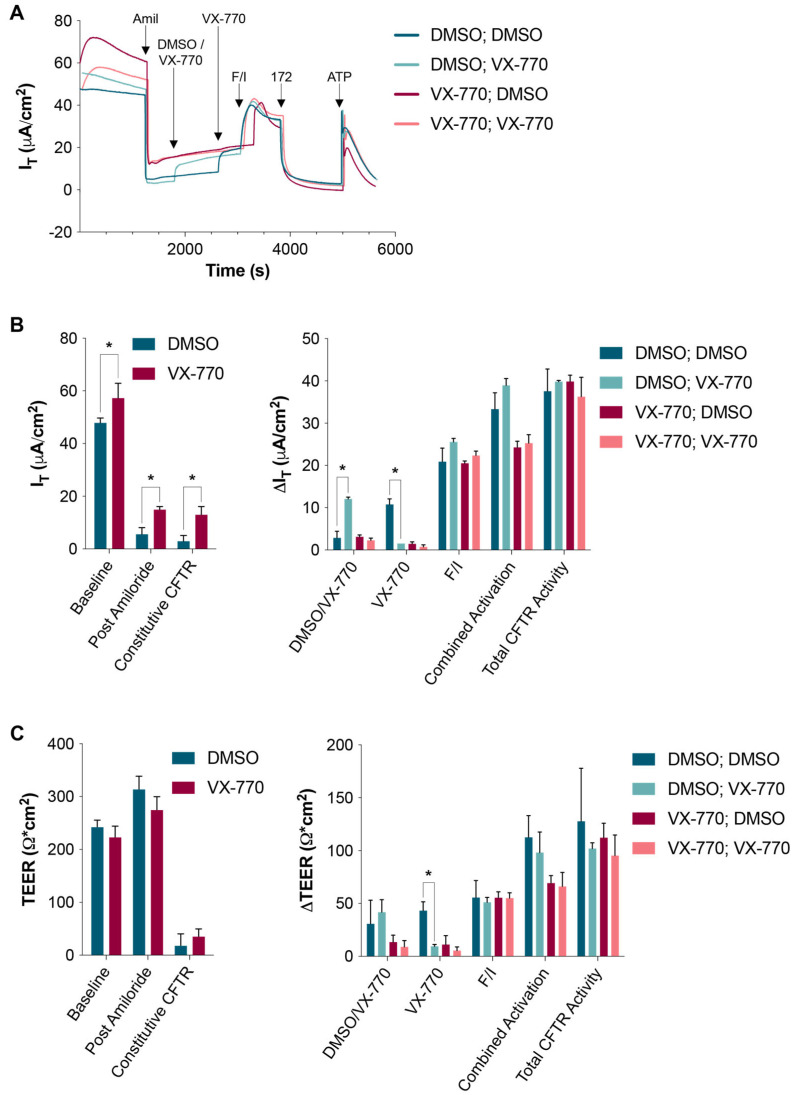
Acute and chronic exposures to saturating concentrations of VX-770 resulted in the same impact on CFTR-mediated currents. For 24 h before analysis in an Ussing chamber, HNECs were exposed to either DMSO or 0.1 μM VX-770 in culture media. (**A**) The HNECs were treated as described for Figure 1, with an additional 1 μM VX-770 treatment (applied to all cultures) prior to F/I to demonstrate saturation and to saturate all the cultures. In the legend, the groups are defined with the first descriptor denoting the chronic treatment (prior to the analysis) and the second descriptor denoting the initial post-amiloride acute treatment during the analysis. (**B**,**C**) The transepithelial current (I_T_) and resistance (TEER) values at baseline, after amiloride treatment; values representing the amount of constitutive CFTR are presented in the left panels, and changes in the values due to exposure to the listed compounds are presented in the right panels. * *p* < 0.05. For the data in the left panels, *n* = 5–7 per group, and in the right panels, *n* = 2–4 per group.

**Figure 3:**
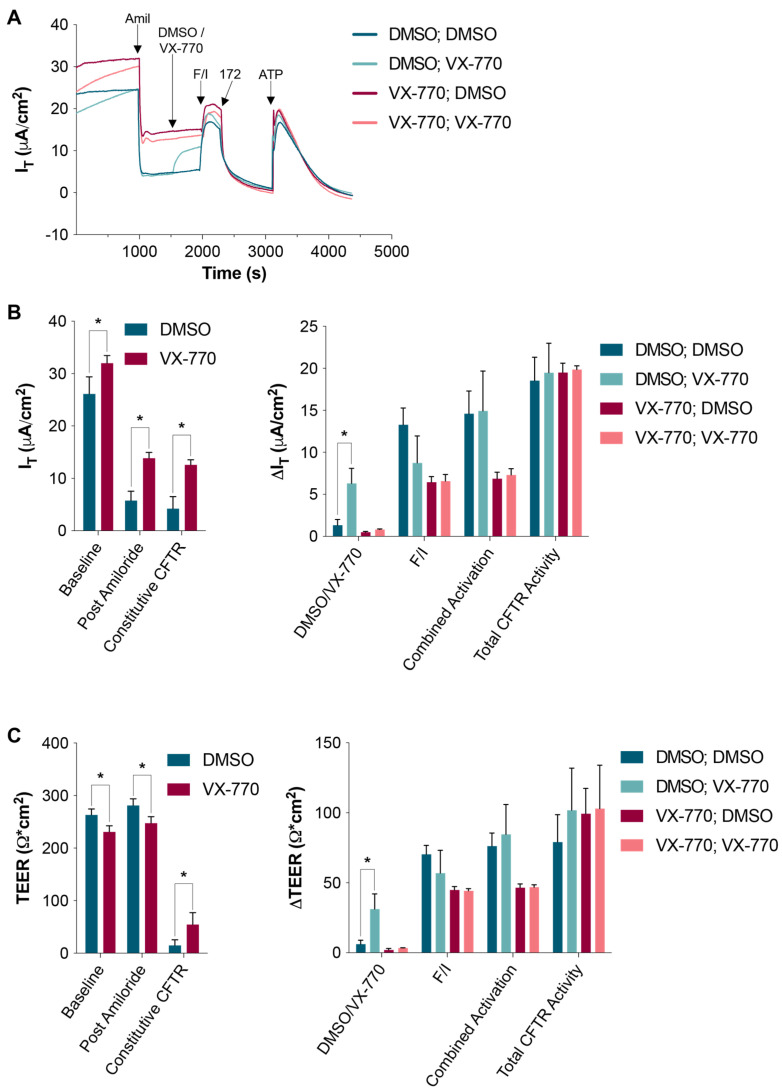
Short-term apical exposure to VX-770 resulted in a chronic impact on CFTR-mediated ion transport. The HNECs were exposed to DMSO or VX-770 during the analysis in an Ussing chamber (see Figure 1), rinsed and returned to the culture, and then re-analyzed 24 h later. (**A**) The HNECs were treated as described for Figure 1, with the groups defined as for Figure 2. (**B**,**C**) The values (left panels) or changes in values (right panels) were quantified. * *p* < 0.05. For the data in the left panels, *n* = 6 per group, and in the right panels, *n* = 3 per group.

**Figure 4:**
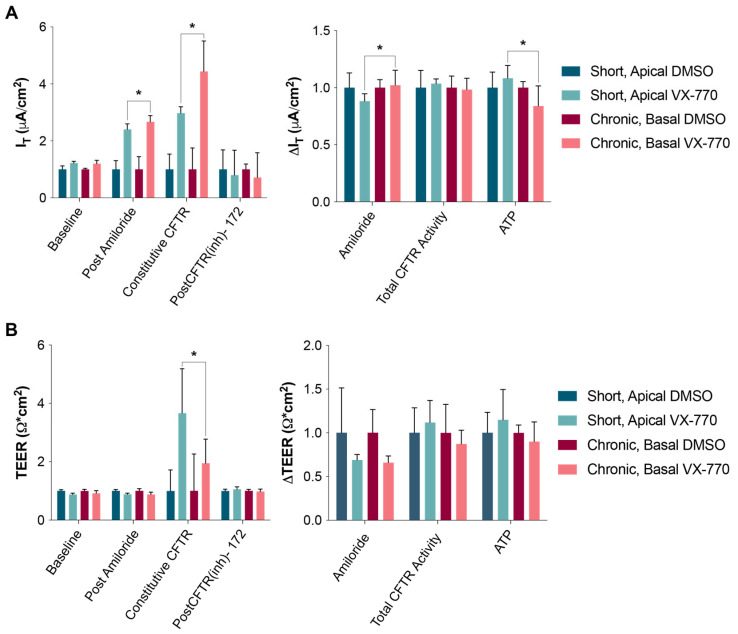
Acute and chronic VX-770 exposures resulted in a similar enduring impact on CFTR-mediated ion transport. The data from the experiments depicted in Figure 2 and Figure 3 were normalized to the internal DMSO controls. The values (left panels) or changes in values (right panels) in the current (**A**) and resistance (**B**) are shown. The “chronic, basal” values were derived from the data shown in Figure 2, and the “short, apical” values were derived from the data shown in Figure 3. * *p* < 0.05. *n* = 5–7 per group.

The electrophysiological analyses were performed in an Ussing chamber (Physiologic Instruments, Reno, NV, USA) under current clamp (0 μA) conditions with intermittent current pulsing (200 ms pulses at +/– 5 μA). The transepithelial current (I_T_), resistance (TEER), and potential difference (TEPD) values were continuously monitored. The I_T_ and TEER values are shown in all the figures, and the TEPD values for Figures 1, 2, 3 and 4 are shown in Supplemental Figures S1, S2, S3 and S4, respectively. The cells were symmetrically bathed in a modified Ringer’s solution (120 mM NaCl, 10 mM D-glucose, 3.3 mM KH_2_PO_4_, 0.83 mM K_2_HPO_4_, 1.2 mM MgCl_2_, 1.2 mM CaCl_2_, 25 mM NaHCO_3_, and a pH of 7.4), maintained at 37 °C, and gassed with 5% CO_2_/95% O_2_. During the analysis, the following concentrations of compounds were sequentially applied: apical 10 μM amiloride (#J62168, Alfa Aesar); apical 1 μM VX-770; 20 μM forskolin (#11018, Cayman Chemicals, Ann Arbor, MI, USA)/100 μM IBMX (#I5879, Sigma-Aldrich) to both chambers; apical 20 μM CFTR (inh)-172 (Cystic Fibrosis Foundation Therapeutics CFTR Compound Program); and apical 100 μM ATP (#A26209, Sigma-Aldrich).

### 2.3. Data Analysis and Statistics 

The results for each experimental data set depict the data generated for cultures from a single donor and are representative of the data obtained from multiple unique donors. For the drug responses, all changes in values are absolute values to allow for differences amongst the groups to be more clearly displayed; the exceptions are the TEPD values for baseline and post-amiloride, shown in Supplemental Figures S2 and S3. The “combined activation” values were calculated by subtracting the value immediately before the first post-amiloride treatment from the maximum value obtained with the forskolin/IBMX (F/I) stimulation. The “total CFTR activity” values were calculated by subtracting the value after the CFTR (inh)-172 treatment from the maximum value obtained with F/I stimulation.

To test the distribution of the data sets for normality, the baseline current values and the values of the changes in the current in response to amiloride from technical replicates were analyzed using the Shapiro-Wilk normality test (α = 0.05). Only one group did not pass the normality test; this group contained a significant outlier, as determined by ROUT testing, and after removal of this data set from the study, the data in this group were found to be normally distributed. For comparisons of groups, *t*-tests were performed, with resulting *p*-values of less than or equal to 0.05 considered significant. All the statistical analyses were performed using Prism 10 (v10.3.1, GraphPad Software).

## 3. Results

### 3.1. Acute and 24-h Exposures to VX-770 Similarly Increase CFTR-Mediated Currents

The differentiated human nasal epithelial cell (HNEC) cultures demonstrated typical responses to stimuli, including a decrease in the current upon the inhibition of the epithelial sodium channel (ENaC) using amiloride and an increase in the current upon the stimulation of calcium-activated chloride channels (CaCCs) through ATP (Figure 1A). In this and later figures, “combined activation” is defined as the difference between the maximal value reached with forskolin/IBMX (F/I) stimulation and the value before the addition of the first treatment after amiloride. The “total CFTR activity” is defined as the difference between the maximal value reached with F/I stimulation and the value obtained after CFTR (inh)-172-mediated inhibition; therefore, in addition to the CFTR activity induced by the addition of F/I, this value includes the constitutive activity that we define as the CFTR activity due to phosphorylation through endogenous cellular pathways (also referred to as “spontaneous” or “basal” CFTR activity [23,25,26]). As previously demonstrated by our group [27], non-CF epithelia respond to VX-770 in the absence of CFTR-activating compounds (e.g., forskolin and IBMX), but VX-770 does not increase the total CFTR activity (Figure 1B,C). As a result, the response to F/I is smaller in HNECs previously exposed to VX-770.

In a separate experiment, HNECs were treated with either DMSO or 0.1 μM VX-770 in the basal media for 24 h prior to the analysis. Upon acute exposure to 1 μM VX-770 during the analysis, the DMSO-treated cultures responded as normal, while the VX-770-treated cultures showed no response, demonstrating that the 24-h exposure at 0.1 μM was sufficient for the saturation of the CFTR (Figure 2). A subsequent acute dosing with another 1 μM VX-770 demonstrated that the initial acute dose of VX-770 resulted in the saturation of the CFTR in cultures that were naïve to VX-770. The increase in CFTR-mediated ion transport induced by the chronic exposure to VX-770 was evident when examining the current values after treatment with amiloride and in the values of constitutive currents (Figure 2B, left panel). However, even with these longer exposures, there was no change in the total CFTR activity (Figure 2B, right panel), suggesting that there are no differences in CFTR expression caused by chronic VX-770 treatment. When examining the TEER values, it can be observed that there was a slight increase in resistance upon the exposure to DMSO in the Ussing chamber (Figure 2C, right panel), and this increase was most likely responsible for the lack of significant differences in the TEER, as shown in the left panel of Figure 2C.

After collecting the data depicted in Figure 1, the cultures utilized to generate these results were thoroughly rinsed with warm PBS, and then returned to the incubator with fresh media containing antibiotics and antifungals. A total of 24 h later, the HNECs were re-analyzed as before (Figure 3). Importantly, the HNECs that were exposed to 1 μM VX-770 apically while in the Ussing chamber (for approximately 45 min) behaved similarly to the HNECs treated basally with 0.1 μM VX-770 for 24 h (Figure 2), with the CFTR being saturated after either exposure. The data shown in Figure 2 and Figure 3 were then normalized to the DMSO controls for their specific data sets, and are displayed in Figure 4. This presentation highlights the similarities between the impacts of the short, apical exposure and the chronic, basal exposure to VX-770 (Figure 4, left panels). Also shown in the normalized data sets are the responses to amiloride, the total CFTR activities, and the responses to ATP, demonstrating a lack of impact of VX-770 exposure on these properties. Interestingly, the chronic, basal 0.1 μM VX-770 exposure resulted in a slightly larger increase in the constitutive CFTR-mediated currents (and, therefore, the post-amiloride current) as compared to the short, apical 1 μM exposure.

**Figure 5:**
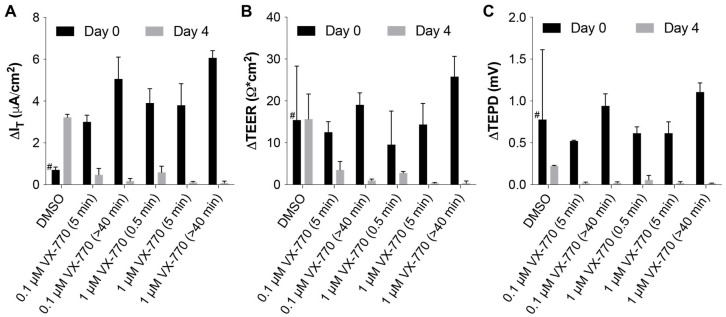
A preliminary experiment demonstrated CFTR saturation with a short exposure time and low concentrations of VX-770. During an initial analysis in an Ussing chamber (Day 0), HNECs were exposed to DMSO or VX-770 for various time periods, and were then returned to the culture and re-analyzed 4 days later; the groups are labeled according to the treatment regimen at Day 0. Changes in the response to DMSO or the indicated concentration of VX-770 for the I_T_ (**A**), TEER (**B**), and transepithelial potential difference (TEPD) (**C**) are given. The term “>40 min” denotes that VX-770 was in the apical chamber for the duration of the analysis, as depicted in Figure S5. For 0.5 and 5 min exposures, the values were calculated after 15 min from the initiation of exposure to allow the HNECs to recover from washing. *n* = 2 per group. A total of 4 days later (Day 4), all the cultures were exposed to 1 μM VX-770 and the responses were quantified. # The treatment in the “DMSO” group was DMSO on Day 0.

A preliminary experiment was performed to examine the acute and chronic consequences of exposures of HNECs to VX-770 for shorter time periods and at lower concentrations (Figure 5 and Figure S5). During the Ussing chamber analysis, the HNECs were treated in duplicate with VX-770 for time periods as short as 0.5 min and at concentrations of either 0.1 or 1 μM (Figure 5, “Day 0” values). For the 0.5 and 5 min exposures, the buffer in the apical chamber was replaced after the exposure period with fresh buffer containing amiloride, and then this rinse process was repeated two additional times. After this analysis, the HNECs were returned to the culture and then re-analyzed after a period of 4 days (Figure 5, “Day 4” values). The “Day 0” responses to VX-770 indicate that 0.1 μM VX-770 induces a similar amount of current change as 1 μM, and that exposure to 1 μM VX-770 for 0.5 min induces a similar amount of current change as a 5 min exposure. Importantly, the response to 1 μM VX-770 4 days later was almost completely absent from the cultures that were exposed to VX-770 on Day 0, regardless of the dose or length of the exposure, but there was a strong response in the cultures that were naïve to VX-770. These results demonstrate that, even with lower-exposure doses and shorter periods of exposure as well as an extended amount of time before the re-analysis, the CFTR in these HNECs remains saturated with VX-770.

### 3.2. Washing the Apical Surface of the HNEC Cultures Increases the Functional Impact of VX-770 Exposure

**Figure 6:**
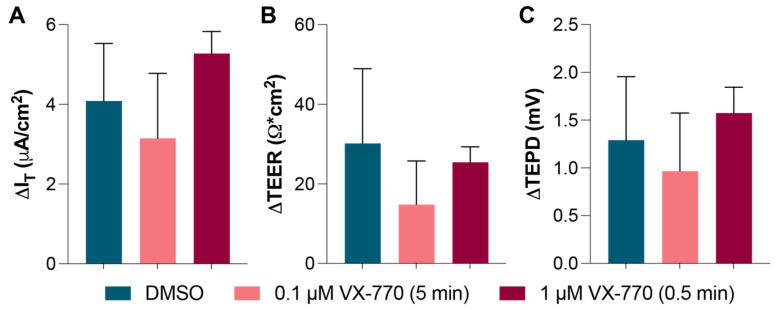
Apical exposure to VX-770 outside of the Ussing chamber did not result in the enduring saturation of the CFTR. HNECs were exposed to the indicated concentration of VX-770 for either 0.5 or 5 min under sterile conditions 4 days prior to the analysis. After stabilizing in the Ussing chamber, the HNECs were treated with amiloride follow by 1 μM VX-770. Changes in the values in response to VX-770 were quantified for the I_T_ (**A**), TEER (**B**), and TEPD (**C**). No significant differences between groups were detected. *n* = 4 per group.

In an experiment attempting to repeat the observations made in the preliminary experiment described in Figure 5, the apical surfaces of HNECs were treated with low doses of VX-770 for short periods of time. In order to increase the efficiency, these treatments were performed outside of the Ussing chamber: VX-770 diluted in HBSS with calcium and magnesium was warmed to 37 °C and applied to the apical surface in a volume of 100 μL under sterile conditions. For cultures exposed for 5 min, the HNECs were returned to the incubator during the exposure time. Following the exposure to VX-770, the cells were rinsed twice with 200 μL of fresh HBSS. A total of 4 days after these short exposures, the HNECs were analyzed (Figure 6 and Figure S6). In contrast with the results from the preliminary experiment (Figure 5), there was no decrease in the VX-770 response in the cultures that had been previously exposed to VX-770. 

**Figure 7:**
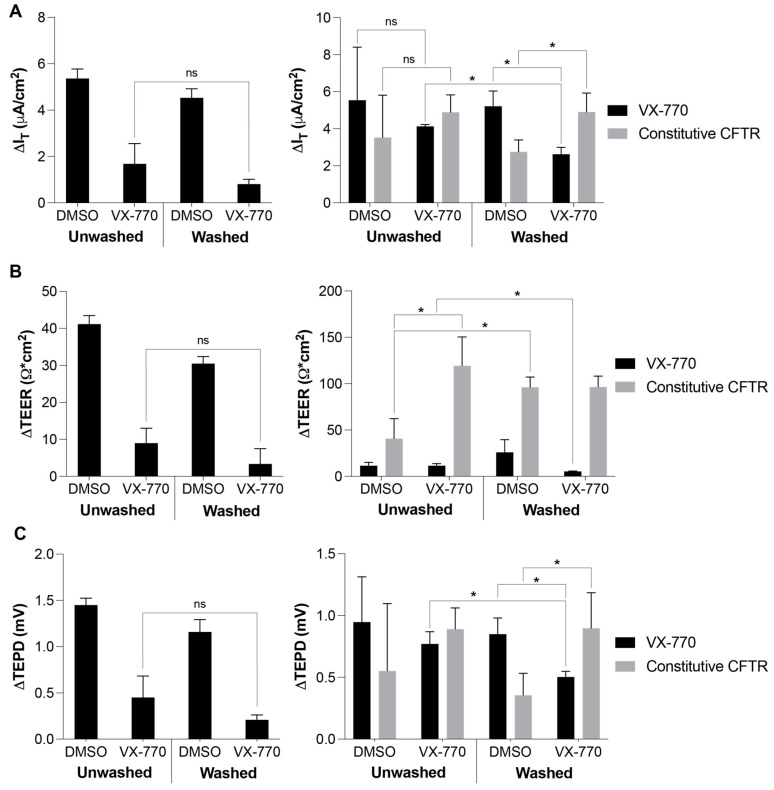
Apical washing prior to VX-770 exposure increases VX-770 interactions with the CFTR. HNECs underwent an apical washing procedure (see Methods for details) prior to exposure to DMSO or VX-770, followed by rinsing. A total of 30 min after the exposure, the HNECs were mounted in an Ussing chamber, allowed to stabilize, and then treated sequentially with amiloride, 1 μM VX-770, F/I, and CFTR (inh)-172. The left and right panels depict the data obtained from different donor HNECs. Changes in the values in response to VX-770 were quantified for the I_T_ (**A**), TEER (**B**), and TEPD (**C**). * *p* < 0.05, and “ns” denotes no significance. *n* = 3 per group.

While several factors from alterations in the approach could contribute to these differences, it was first examined whether the “washing” of the apical surface of the HNECs by the circulating buffer in the Ussing chamber provided a means of clearing mucus and cellular products from the surface, thereby making it more accessible to VX-770; mucus may be a considerable barrier in these experiments, given the “stickiness” of both mucus and VX-770. The experimental approach utilized to test this involved performing a series of washes on the apical surface of the HNECs prior to a 5 min exposure to 1 μM VX-770, followed by further washing to remove free VX-770, after which the HNECs were incubated for 25 min before the analysis in an Ussing chamber to examine the response to acute VX-770 (Figure 7 and Figure S7). In the HNECs from the first donor utilized for this test (Figure 7, left panels), washing the apical surface prior to the initial exposure to VX-770 did not increase the interaction between VX-770 and CFTR (as measured through a decrease in the subsequent response to acute VX-770 during the analysis) compared to the degree of interaction in the unwashed HNECs. However, a significant decrease in the VX-770 response in the Ussing chamber was observed in HNECs that were previously exposed to VX-770 without washing (i.e., processing was the same as for the HNECs utilized in Figure 5, during which no significant decrease was observed). In repeating this experiment with HNECs from a second donor (Figure 7, right panels), we did not observe a significant decrease in the VX-770 response in cultures that were not apically washed prior to VX-770 exposure, but we did observe a decrease in the current response to VX-770 in those that were washed. Additionally, in the HNECs washed prior to VX-770 exposure, we observed the same increase in constitutive CFTR currents as that detected in previous experiments, in which the CFTR appeared to be saturated with VX-770 (Figure 7A, gray bars). Interestingly, while these same impacts from apical washing were evident in the changes in the TEPD (Figure 7C), they were not apparent in the changes in the TEER (Figure 7B); this may be due, in part, to the impact of the apical wash itself on the properties of the HNECs (Figure 7B, gray bars). It is possible that donor-to-donor variations in the amount of secreted material on the apical surface contributed to the observed differences in the outcomes between Figure 6 and the left and right panel data in Figure 7.

## 4. Discussion

The results presented in this study support previous reports demonstrating that the functional impact of VX-770 on CFTR-mediated ion transport requires low concentrations and occurs rapidly. No differences in the electrophysiological outcome were observed between the cultures exposed apically to VX-770 while mounted in an Ussing chamber and the cultures exposed basally to media containing VX-770 during normal incubation. Importantly, the effects of VX-770 endured for more than 4 days after a short-term, low-concentration apical exposure during the analysis in an Ussing chamber. Additionally, secreted cellular products on the apical surface appeared to inhibit the binding of VX-770 to CFTR.

In a similar study performed by another team [21], it was observed that primary airway epithelial cultures incubated with 1 μM VX-770 for 14 days continuously accumulated VX-770 intracellularly. From day 7 to day 14, they observed a decrease in the forskolin/IBMX and subsequent CFTR (inh)-172 responses; while the decrease in the CFTR (inh)-172 response suggests a decrease in the total functional protein expression, it is possible that the constitutive activity of the CFTR in their cultures remained stable. Upon the removal of VX-770 from the culture media at day 14, they did not observe any substantial decrease in the levels of VX-770 over the 14-day period following withdrawal; we expect that VX-770 remained intracellularly post-exposure in our cultures as well, including those exposed only apically at a lower concentration and for a dramatically shorter period of time. All the experiments in their study were performed in primary human bronchial epithelial cells obtained from people with CF that were homozygous for the F508del CFTR mutation and, as such, treatment with VX-770 was only performed in conjunction with either VX-809 or VX-661, with both being correctors of the F508del CFTR mutation. It is important to note that, after the removal of CFTR modulators from the media, the VX-809 and VX-661 levels rapidly decreased, and as the half-life of CFTR is ~12 h [28], it is possible that the increase in CFTR function (compared to untreated cells) that they observed 14 days after withdrawal was due to the continued presence of VX-770 in the cells. While this report differs in several aspects from our study, the persistent impact of VX-770 may be a common observation.

The results of the present study led to two potential hypotheses: the first hypothesis is that the binding of VX-770 to CFTR is irreversible, resulting in the enduring impact of VX-770 on the CFTR in our cultures. This hypothesis is highly unlikely, as others have measured the dissociation of VX-770 from CFTR using the patch clamp technique, indicating that the binding is reversible [12,13]. Additionally, this hypothesis would require that the half-life of the CFTR protein be much longer than current studies have demonstrated. The second, and favored, hypothesis is that VX-770 rapidly accumulates in the cell, where it remains and can continually bind to freshly synthesized CFTR. This hypothesis is supported by several observations made by others: as mentioned above, VX-770 is detectable by mass spectrometry in cells more than 14 days after cessation of exposure to the compound. Additionally, VX-770 has been found to permeate and remain in a phospholipid bilayer [29,30,31]. This property may contribute to the intracellular longevity of the compound, and to its ability to readily interact with newly synthesized CFTR protein, despite the fact that VX-770 is commonly believed to be a “sticky” compound that rapidly adheres to any protein available. This “stickiness” property could be due to the very low solubility of the compound in water [12]; VX-770 may rapidly “stick” to any location that is energetically favorable, perhaps including the plasma membrane. Our second hypothesis is also supported by the very high potency of the VX-770 compound [12]. Taken together, our favored hypothesis is that, being poorly soluble in aqueous solutions, VX-770 rapidly inserts into the plasma membrane, where it stably persists and accumulates during exposures. While in the membrane, it can potently interact with CFTR, resulting in an increase in the open probability of the channel.

To our knowledge, no one has performed a washout study to examine the longevity of VX-770 and/or VX-770-mediated effects in the airways of people undergoing a VX-770-containing therapy. However, VX-770 washout has been studied in the context of ferrets [32]. In the model reported in that study, CFTR^G551D/-^ ferrets were exposed to VX-770 in utero beginning on embryonic day 28, and VX-770 was then withdrawn at various times after birth. While the group performing this study did not measure the VX-770 levels in the airways, they did monitor the clinical outcomes. Interestingly, the consequences of VX-770 withdrawal were highly variable from animal to animal, but overall, the onset of symptoms was more rapid when VX-770 was withdrawn at a younger age. For example, after the withdrawal of VX-770 at 14 days of age, some animals met euthanasia criteria within weeks (although others survived past 250 days of age). When withdrawing VX-770 at 267 days of age, it took a minimum of 51 days for culturable bacteria obtained by serial bronchoscopies to appear in the lungs. It is possible that the impact of VX-770 is more severe during the developmental period, but it may also be possible that the rapid growth of the animals at 14 days of age contributes to the cellular dilution of the accumulated VX-770, resulting in a more immediate onset of symptoms. Additional experiments may shed light on the mechanisms responsible for these age-related differences in the onset of symptoms.

Further studies in vitro and in animal models should provide insights into the length of persistence of VX-770 and VX-770-mediated effects in airway cells. Two of the observations made in the current study may inform the design of future experiments. First, it was demonstrated that the saturation of CFTR with VX-770 is achievable with short exposures to low concentrations of VX-770. Second, a novel and effective measurement of the continued impact of VX-770 on the CFTR can be obtained by examining the response to the re-exposure to VX-770 during an electrophysiological analysis; if the CFTR is saturated with VX-770, then no response to acute exposure will be observed, and partial saturation results in a decrease in the magnitude of the response to VX-770.

The results of the experiments reported herein show that brief exposures of differentiated primary airway epithelial cultures to nanomolar concentrations of VX-770 induce an increase in CFTR-mediated currents, and the interaction between VX-770 and CFTR following exposure continues over extended time periods. Overall, given the continued development of therapeutics to treat CF as well as the potential expansion of CFTR modulators to treat diseases other than CF, it may be valuable to determine the longevity of the impact of VX-770 on the function of the CFTR in the airway epithelium in vivo. 

## Data Availability

The data supporting the reported results are available from the corresponding author upon request.

## Supplementary Materials

The following supporting information can be downloaded at: https://www.mdpi.com/article/10.3390/biom14111378/s1, Figure S1: Changes in the transepithelial potential difference (TEPD) values corresponding to the data shown in Figure 1; Figure S2: Transepithelial potential difference (TEPD) values (left panel) and changes in the TEPD values (right panel) corresponding to the data shown in Figure 2; Figure S3: Transepithelial potential difference (TEPD) values (left panel) and changes in the TEPD values (right panel) corresponding to the data shown in Figure 3; Figure S4: Transepithelial potential difference (TEPD) values (left panel) and changes in the TEPD values (right panel) corresponding to the data shown in Figure 4; Figure S5: Transepithelial current (IT) traces corresponding to the data quantified in Figure 5A; Figure S6: Transepithelial current (IT) traces corresponding to the data quantified in Figure 6A; Figure S7: Transepithelial current (IT) traces corresponding to the data shown in Figure 7A.

## References

[1] Van Goor F., Hadida S., Grootenhuis P.D., Burton B., Cao D., Neuberger T., Turnbull A., Singh A., Joubran J., Hazlewood A. (2009). Rescue of CF airway epithelial cell function in vitro by a CFTR potentiator, VX-770. Proc. Natl. Acad. Sci. USA.

[2] Van Goor F., Yu H., Burton B., Hoffman B.J. (2014). Effect of ivacaftor on CFTR forms with missense mutations associated with defects in protein processing or function. J. Cyst. Fibros..

[3] Yu H., Burton B., Huang C.J., Worley J., Cao D., Johnson J.P., Urrutia A., Joubran J., Seepersaud S., Sussky K. (2012). Ivacaftor potentiation of multiple CFTR channels with gating mutations. J. Cyst. Fibros..

[4] Accurso F.J., Rowe S.M., Clancy J.P., Boyle M.P., Dunitz J.M., Durie P.R., Sagel S.D., Hornick D.B., Konstan M.W., Donaldson S.H. (2010). Effect of VX-770 in persons with cystic fibrosis and the G551D-CFTR mutation. N. Engl. J. Med..

[5] Ramsey B.W., Davies J., McElvaney N.G., Tullis E., Bell S.C., Drevinek P., Griese M., McKone E.F., Wainwright C.E., Konstan M.W. (2011). A CFTR potentiator in patients with cystic fibrosis and the G551D mutation. N. Engl. J. Med..

[6] Davies J.C., Wainwright C.E., Canny G.J., Chilvers M.A., Howenstine M.S., Munck A., Mainz J.G., Rodriguez S., Li H., Yen K. (2013). Efficacy and safety of ivacaftor in patients aged 6 to 11 years with cystic fibrosis with a G551D mutation. Am. J. Respir. Crit. Care Med..

[7] Guimbellot J., Solomon G.M., Baines A., Heltshe S.L., VanDalfsen J., Joseloff E., Sagel S.D., Rowe S.M., Investigators G.O. (2019). Effectiveness of ivacaftor in cystic fibrosis patients with non-G551D gating mutations. J. Cyst. Fibros..

[8] De Boeck K., Munck A., Walker S., Faro A., Hiatt P., Gilmartin G., Higgins M. (2014). Efficacy and safety of ivacaftor in patients with cystic fibrosis and a non-G551D gating mutation. J. Cyst. Fibros..

[9] Pyle L.C., Ehrhardt A., Mitchell L.H., Fan L., Ren A., Naren A.P., Li Y., Clancy J.P., Bolger G.B., Sorscher E.J. (2011). Regulatory domain phosphorylation to distinguish the mechanistic basis underlying acute CFTR modulators. Am. J. Physiol. Lung Cell. Mol. Physiol..

[10] Eckford P.D., Li C., Ramjeesingh M., Bear C.E. (2012). Cystic fibrosis transmembrane conductance regulator (CFTR) potentiator VX-770 (ivacaftor) opens the defective channel gate of mutant CFTR in a phosphorylation-dependent but ATP-independent manner. J. Biol. Chem..

[11] Jih K.Y., Hwang T.C. (2013). Vx-770 potentiates CFTR function by promoting decoupling between the gating cycle and ATP hydrolysis cycle. Proc. Natl. Acad. Sci. USA.

[12] Csanady L., Torocsik B. (2019). Cystic fibrosis drug ivacaftor stimulates CFTR channels at picomolar concentrations. eLife.

[13] Yeh H.I., Qiu L., Sohma Y., Conrath K., Zou X., Hwang T.C. (2019). Identifying the molecular target sites for CFTR potentiators GLPG1837 and VX-770. J. Gen. Physiol..

[14] Laselva O., Qureshi Z., Zeng Z.W., Petrotchenko E.V., Ramjeesingh M., Hamilton C.M., Huan L.J., Borchers C.H., Pomes R., Young R. (2021). Identification of binding sites for ivacaftor on the cystic fibrosis transmembrane conductance regulator. iScience.

[15] Langron E., Prins S., Vergani P. (2018). Potentiation of the cystic fibrosis transmembrane conductance regulator by VX-770 involves stabilization of the pre-hydrolytic, O(1) state. Br. J. Pharmacol..

[16] Guimbellot J.S., Ryan K.J., Anderson J.D., Parker K.L., Victoria Odom L., Rowe S.M., Acosta E.P. (2022). Plasma and cellular ivacaftor concentrations in patients with cystic fibrosis. Pediatr. Pulmonol..

[17] Guimbellot J.S., Ryan K.J., Anderson J.D., Liu Z., Kersh L., Esther C.R., Rowe S.M., Acosta E.P. (2020). Variable cellular ivacaftor concentrations in people with cystic fibrosis on modulator therapy. J. Cyst. Fibros..

[18] Pigliasco F., Cafaro A., Stella M., Baiardi G., Barco S., Pedemonte N., D’Orsi C., Cresta F., Casciaro R., Castellani C. (2023). Simultaneous Quantification of Ivacaftor, Tezacaftor, and Elexacaftor in Cystic Fibrosis Patients’ Plasma by a Novel LC-MS/MS Method. Biomedicines.

[19] Matthes E., Goepp J., Carlile G.W., Luo Y., Dejgaard K., Billet A., Robert R., Thomas D.Y., Hanrahan J.W. (2016). Low free drug concentration prevents inhibition of F508del CFTR functional expression by the potentiator VX-770 (ivacaftor). Br. J. Pharmacol..

[20] Cholon D.M., Quinney N.L., Fulcher M.L., Esther C.R., Das J., Dokholyan N.V., Randell S.H., Boucher R.C., Gentzsch M. (2014). Potentiator ivacaftor abrogates pharmacological correction of DeltaF508 CFTR in cystic fibrosis. Sci. Transl. Med..

[21] Guhr Lee T.N., Cholon D.M., Quinney N.L., Gentzsch M., Esther C.R. (2020). Accumulation and persistence of ivacaftor in airway epithelia with prolonged treatment. J. Cyst. Fibros..

[22] Shaughnessy C.A., Zeitlin P.L., Bratcher P.E. (2021). Elexacaftor is a CFTR potentiator and acts synergistically with ivacaftor during acute and chronic treatment. Sci. Rep..

[23] Nick H.J., Zeitlin P.L., Yadav S., Bratcher P.E. (2021). Measurements of spontaneous CFTR-mediated ion transport without acute channel activation in airway epithelial cultures after modulator exposure. Sci. Rep..

[24] Goldfarbmuren K.C., Jackson N.D., Sajuthi S.P., Dyjack N., Li K.S., Rios C.L., Plender E.G., Montgomery M.T., Everman J.L., Bratcher P.E. (2020). Dissecting the cellular specificity of smoking effects and reconstructing lineages in the human airway epithelium. Nat. Commun..

[25] Becq F., Mirval S., Carrez T., Leveque M., Billet A., Coraux C., Sage E., Cantereau A. (2022). The rescue of F508del-CFTR by elexacaftor/tezacaftor/ivacaftor (Trikafta) in human airway epithelial cells is underestimated due to the presence of ivacaftor. Eur. Respir. J..

[26] Shaughnessy C.A., Zeitlin P.L., Bratcher P.E. (2022). Net benefit of ivacaftor during prolonged tezacaftor/elexacaftor exposure in vitro. J. Cyst. Fibros..

[27] Bratcher P.E., Yadav S., Shaughnessy C.A., Thornell I.M., Zeitlin P.L. (2020). Effect of apical chloride concentration on the measurement of responses to CFTR modulation in airway epithelia cultured from nasal brushings. Physiol. Rep..

[28] Varga K., Goldstein R.F., Jurkuvenaite A., Chen L., Matalon S., Sorscher E.J., Bebok Z., Collawn J.F. (2008). Enhanced cell-surface stability of rescued DeltaF508 cystic fibrosis transmembrane conductance regulator (CFTR) by pharmacological chaperones. Biochem. J..

[29] Baroni D., Zegarra-Moran O., Svensson A., Moran O. (2014). Direct interaction of a CFTR potentiator and a CFTR corrector with phospholipid bilayers. Eur. Biophys. J..

[30] Iazzi M., Junor P., Doshi J., Acharya S., Suhring R., Viirre R.D., Gupta G.D. (2023). Synthesis and Evaluation of Ivacaftor Derivatives with Reduced Lipophilicity. ACS Omega.

[31] Chin S., Hung M., Won A., Wu Y.S., Ahmadi S., Yang D., Elmallah S., Toutah K., Hamilton C.M., Young R.N. (2018). Lipophilicity of the Cystic Fibrosis Drug, Ivacaftor (VX-770), and Its Destabilizing Effect on the Major CF-causing Mutation: F508del. Mol. Pharmacol..

[32] Sun X., Yi Y., Yan Z., Rosen B.H., Liang B., Winter M.C., Evans T.I.A., Rotti P.G., Yang Y., Gray J.S. (2019). In utero and postnatal VX-770 administration rescues multiorgan disease in a ferret model of cystic fibrosis. Sci. Transl. Med..

