# Supplementary material for: The Functional Impact of VX-770 on the Cystic Fibrosis Transmembrane Conductance Regulator Is Enduring and Increases the Constitutive Activity of This Channel in Primary Airway Epithelia Generated from Healthy Donors
Source: Biomolecules. 2024 Oct 29;14(11):1378. doi: 10.3390/biom14111378 (PMC11591604; doi:10.3390/biom14111378)
Supplement: Supplementary file 1 [file biomolecules-14-01378-s001.zip › Supplemental Figure S5_V3.pdf]

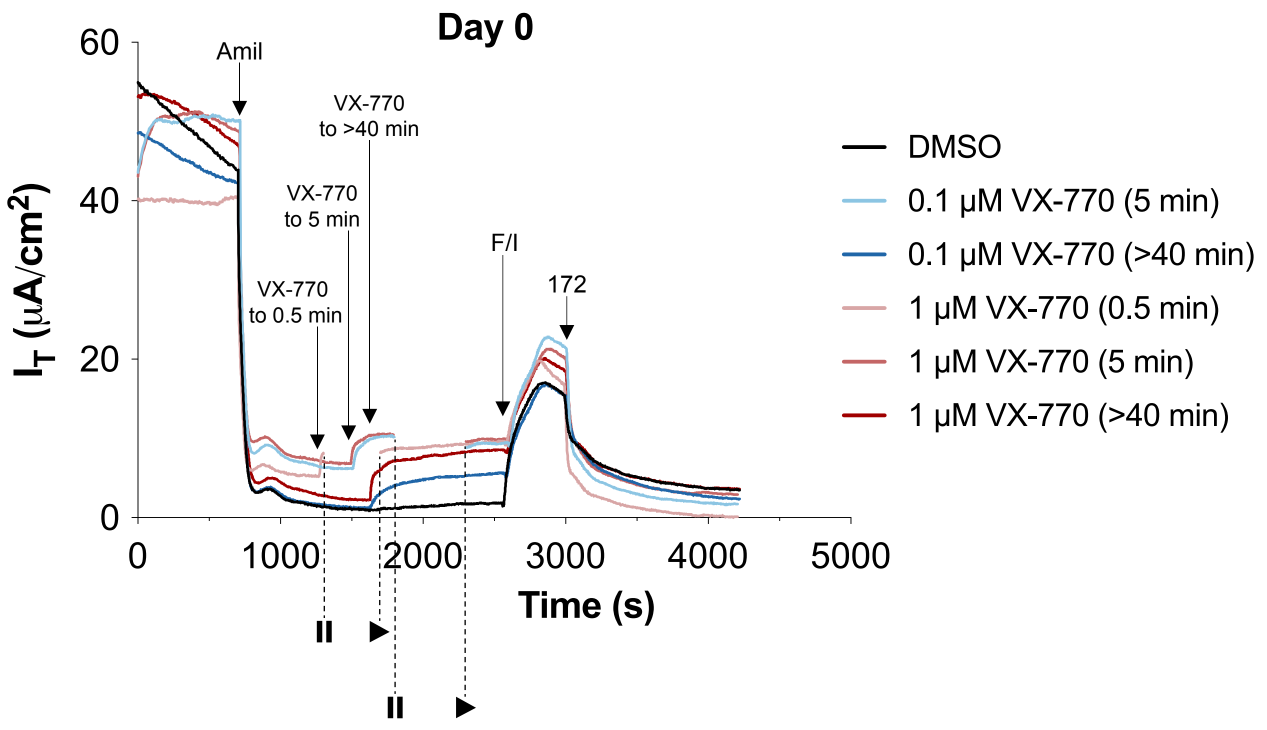

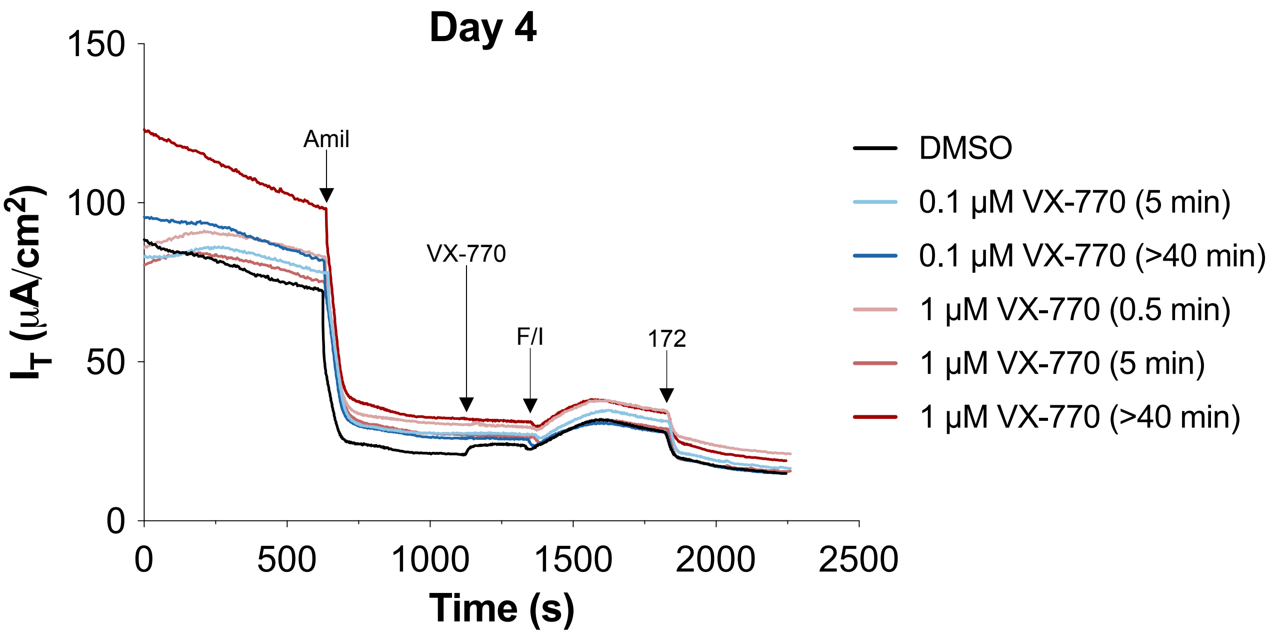

Supplemental Figure S5. Transepithelial current (I_T) traces corresponding to data quantified in Figure 5A. The symbols **II** and ▶ denote the times at which data collection was paused and resumed, respectively, over the period during which the chambers and cultures were being washed.
