# Supplementary material for: The Functional Impact of VX-770 on the Cystic Fibrosis Transmembrane Conductance Regulator Is Enduring and Increases the Constitutive Activity of This Channel in Primary Airway Epithelia Generated from Healthy Donors
Source: Biomolecules. 2024 Oct 29;14(11):1378. doi: 10.3390/biom14111378 (PMC11591604; doi:10.3390/biom14111378)
Supplement: Supplementary file 1 [file biomolecules-14-01378-s001.zip › Supplemental Figure S7.pdf]

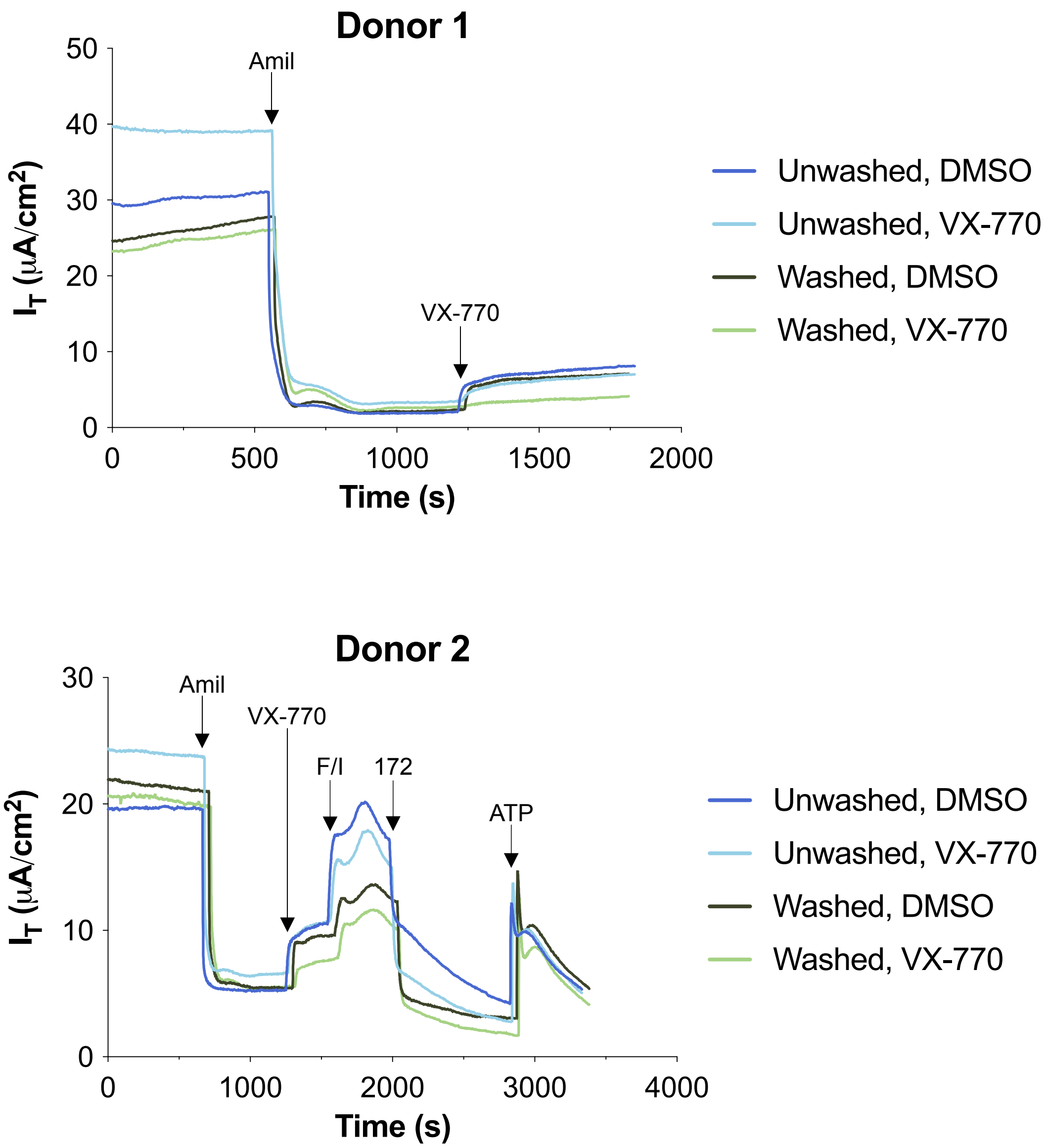

Supplemental Figure S7. Transepithelial current (I_T) traces corresponding to data shown in Figure 7A.
